# Association of Lipid Profile in Pregnancy with Preeclampsia, Gestational Diabetes Mellitus, and Preterm Delivery

**DOI:** 10.7759/cureus.1420

**Published:** 2017-07-03

**Authors:** Babita Ghodke, Raghuram Pusukuru, Varshil Mehta

**Affiliations:** 1 Department of Medicine, MGM Medical College, Navi Mumbai, India; 2 Department of Internal Medicine, MGM Medical College, Navi Mumbai, India

**Keywords:** lipid profile, gestational diabetes mellitus, preterm, preeclampsia, pregnancy-related disease

## Abstract

Introduction

During the last two trimesters of pregnancy, glucose is spared (for the foetus), while the concentration of fatty acids in plasma increases, which can create complications like preeclampsia, gestational diabetes mellitus (GDM), and preterm delivery.

Aim

To study the association of serum lipid levels during the second and third trimesters with the development of pregnancy-associated diseases, such as preeclampsia, GDM, and preterm delivery.

Methods and Materials

The present study was carried out at MGM Hospital, Navi Mumbai, India. Two hundred antenatal cases from October 2012 to October 2014 were enrolled after giving an informed consent. A lipid profile was recorded for each subject and was later accessed. The lipid profile of the subjects having either GDM, preterm, or preeclampsia was further used to find an association with the individual disorders. Statistical analyses were performed using Statistical Package for Social Sciences (SPSS) version 20 (IBM SPSS Statistics, Armonk, NY). All reported p-values are two-tailed, and confidence intervals were calculated at the 95% level.

Results

In preeclamptic patients, the mean systolic blood pressure was 151.40 mm/Hg and the mean diastolic blood pressure was 74.03 mm/Hg in the third trimester. In preeclamptic patients, the mean serum triglyceride levels in the second trimester were 204.00 mg/dl, while the mean was 243.20 mg/dl in the third trimester. In GDM patients, the mean serum triglyceride was 214.33 mg/dl in the second trimester, while it was 230.50 mg/dl in the third trimester. In patients with preterm, the mean triglycerides levels were 212.83 mg/dl and 240.16 mg/dl in the second and third trimesters, respectively. In preeclamptic patients, the mean serum cholesterol levels in the second trimester were 210 mg/dl, while in the third trimester, the mean was 243.60 mg/dl. In GDM patients, the mean serum cholesterol was 223.50 mg/dl and 242.83 mg/dl in the second and third trimester, respectively. The mean cholesterol levels in patients with preterm in second and third trimesters were 213.33 mg/dl and 243.66 mg/dl, respectively. Out of the total 200 patients, 168 had no complications, while 20 (10%) had preeclampsia, six (3%) had gestational diabetes mellitus, and the other six {3%} had preterm deliveries.

Conclusion

An association between maternal early pregnancy triglyceridaemia and the subsequent risk of preeclampsia, gestational diabetes, and preterm deliveries was observed. The occurrence of preeclampsia, gestational diabetes, and preterm deliveries cannot be predicted based on the values of serum cholesterol, high-density lipoprotein cholesterol (HDL-C), low-density lipoprotein cholesterol (LDL-C), and very-low-density lipoprotein cholesterol (VLDL-C). Hence, estimation of lipid profile is strongly recommended during pregnancy to prevent the deleterious effect of hyperlipidaemia associated with pregnancy.

## Introduction

Pregnancy not only demands more metabolic fuels but also causes an alteration in hormonal levels, which may cause few changes in lipid profile during pregnancy [1].

Patients with a pregnancy associated with previous gestational diabetes mellitus (GDM) have an increased risk of developing Type 2 diabetes or cardiovascular diseases (CVD) in later life [2-3], whereas pregnancies complicated with preeclampsia have the potential to lead to future CVD and metabolic syndrome [4-6]. Hence, it is of utmost importance that these complications should be prevented in the pregnancy itself.

In our previous study (from the same data), we showed that total cholesterol, triglycerides (TG), low-density lipoprotein cholesterol (LDL-C), and very low-density lipoprotein-cholesterol (VLDL-C) increases in the last two trimesters. The increase is even greater in the third trimester when compared to the second. However, high-density lipoprotein cholesterol (HDL-C) levels are decreased in the third trimester when compared to that of the second. The study concluded that estimation of the lipid profile is highly recommended during pregnancy due to its association with high levels of triglycerides, which may lead to preeclampsia, GDM, and preterm delivery [7]. In our other study, we showed a significant negative correlation between thyroid stimulating hormone (TSH) levels and cholesterol and VLDL in the third trimester of the pregnancy [8].

The present study is a continuation of our previous study and is based on the same data. However, unlike our previous study, which was to assess the basic levels of the lipid profile during the pregnancy, this study evaluates the clinical significance of the lipid profile level in pregnancy and its effect on the development of pregnancy-induced diseases, such as GDM, preeclampsia, and preterm.

## Materials and methods

The present study is a continuation of our previous study (same data used) and the material, methods, ethics, inclusion, and exclusion criteria remain the same [7]. This study, however, includes completely different sets of data but are from the same patients. With that said, this study is also a major study and different from our previous publication due to its different aim, results, and conclusion, which was pre-decided to rule out the salome splicing error.

In brief, the present study was conducted at the MGM Hospital, Navi Mumbai, India. Two hundred antenatal cases from October 2012 to October 2014 were enrolled after giving an informed consent. Venous blood samples were collected from all the patients for the measurement of the lipid profile in the 16th week and 32nd week of gestation for analysis. Out of the 200 subjects, 10 developed gestational hypertension late in the third trimester (detected during follow-up after the 32nd week), which was also included.

All pregnant women with a singleton pregnancy with a gestational age of 13-28 weeks, irrespective of parity and gravida, were included. Pregnant women in whom hypertension (HTN) was detected before 14 weeks and those with diseases or complications, such as chronic HTN, obstetric and foetal complications (hydrops foetalis, congenital foetal anomalies), diabetes, renal disorders, and thyroid disorders, were excluded.

Statistical analyses were performed using the Statistical Package for Social Sciences (SPSS) version 20 (IBM SPSS Statistics, Armonk, NY). All reported p-values were two-tailed, and confidence intervals were calculated at the 95% level. The data was presented using frequencies, percentages, and descriptive statistics followed by charts and graphs. The level of significance was set at 5%. All p-values less than 0.05 were treated as significant.

## Results

The mean age of patients was 24.87 years with a standard deviation (SD) of 2.7 years. The minimum age was 18 years and the maximum age was 30 years.

Blood pressure

The mean systolic blood pressure (SBP) in the second trimester was 117.03 mm/Hg with an SD of 10.33 mm/Hg. In the third trimester, it increased to 120.77 with an SD of 14.675 mm/Hg.

In preeclamptic patients, the mean SBP was 151.40 mm/Hg with an SD 6.05 mm/Hg (p =0.00) in the third trimester. There was a highly significant statistical difference in the mean blood pressure values among normal and preeclamptic women in the third trimester.

The mean diastolic blood pressure (DBP) in our study in the third trimester was 72.11 mm/Hg with an SD of 6.88 mm/Hg. In the third trimester, the mean SBP increased to 74.03 mm/Hg with an SD of 8.616 mm/Hg. In preeclamptic patients, the mean was 92.00 mm/Hg with an SD 2.59 mm/Hg (p =0.00). There was a highly significant statistical difference in the mean blood pressure values among normal and preeclamptic women in the third trimester. Out of the 200 patients, 168 had no complications, while 20 had preeclampsia, six had gestational diabetes mellitus (GDM), and the other six had preterm deliveries.

Relation of triglycerides with preeclampsia, GDM, and preterm

The data presented below indicates the 95% confidence interval (CI) in triglyceride levels for patients with outcomes of preeclampsia, GDM, and preterm.

As shown in our previous study, the mean triglyceride level in the second trimester was 188.68 mg/dl with a standard deviation of 20.88 mg/dl. In the third trimester, the mean triglyceride (TG) level increased to 216.78 mg/dl with a standard deviation of 20.09 mg/dl [7].

There was a significant statistical significance observed between serum triglyceride levels with preeclampsia and preterm in both second and third trimesters. There was a statistical significance seen between TG levels and GDM patients in the second trimester; however, it was not seen in the third trimester as shown below in Table 1.

**Table 1:** Association of Triglycerides with Preeclampsia, GDM, and Preterm CI: confidence interval; SD: standard deviation, SEM: standard error of the mean; N: number

Outcome	Trimester	Mean	N	SD	SEM	P Value	95 % CI	
							Lower Bound	Upper Bound
Preeclampsia	Second Trimester	204.00	20	18.90	4.23	0.00	195.71	212.29
	Third Trimester	243.20	20	15.58	3.48	0.00	236.37	250.03
Gestational Diabetes Mellitus	Second Trimester	214.33	6	18.64	7.61	0.00	199.42	229.25
	Third Trimester	230.50	6	17.03	6.95	0.09	216.88	244.12
Preterm	Second Trimester	212.83	6	11.99	4.90	0.00	203.24	222.43
	Third Trimester	240.17	6	7.73	3.16	0.00	233.98	246.35

Relation of cholesterol with preeclampsia, GDM, and preterm

The data shown in Table 2 indicates no statistical significance observed between serum cholesterol and preeclampsia, GDM, and preterm in both second and third trimesters.

**Table 2:** Association of Cholesterol with Preeclampsia, GDM, and Preterm CI: confidence interval; SD: standard deviation, SEM: standard error of the mean; N: number

Outcome	Trimester	N	Mean	SD	SEM	P Value	95% CI	
							Lower Bound	Upper Bound
Preeclampsia	2nd Trimester	20	210.75	24.25	5.42	0.38	199.401	222.10
	3rd Trimester	20	243.60	25.85	5.78	0.84	231.50	255.69
Gestational Diabetes Mellitus	2nd Trimester	6	223.50	25.16	10.27	0.24	197.09	249.90
	3rd Trimester	6	242.83	27.14	11.08	0.98	214.35	271.31
Preterm	2nd Trimester	6	213.33	20.23	8.25	0.86	192.10	234.55
	3rd Trimester	6	243.66	27.200	11.10	0.90	215.12	272.21

Relation of high-density lipoprotein cholesterol (HDL-C) with preeclampsia, GDM, and preterm

In the third trimester, the mean serum HDL-C level in normal patients was 42.78 mg/dl with an SD of 4.31 mg/dl; in preeclamptic patients, the mean was 45.60 mg/dl with an SD of 4.12 mg/dl. Compared to the normal value of 40 - 60 mg/dl, HDL cholesterol level was within normal range in normal pregnancy. In preeclamptic women, the HDL-C level was higher than in normal pregnancy but within normal range.

There was a significant statistical significance observed between serum HDL-C and preeclampsia in both second and third trimesters. However, no statistical significance was observed between HDL-C levels and GDM and preterm.

**Table 3:** Association of HDL Cholesterol with Preeclampsia, GDM, and Preterm CI: confidence interval; SD: standard deviation, SEM: standard error of the mean; N: number

Outcome	Trimester	N	Mean	SD	SEM	P Value	95% CI	
							Lower Bound	Upper Bound
Preeclampsia	2nd Trimester	20	51.80	5.84	1.30	0.04	49.06	54.53
	3rd Trimester	20	45.60	4.12	.92	0.00	43.67	47.52
Gestational Diabetes Mellitus	2nd Trimester	6	52.00	7.07	2.88	0.26	44.57	59.42
	3rd Trimester	6	41.16	7.27	2.97	0.29	33.52	48.80
Preterm	2nd Trimester	6	49.00	6.13	2.50	0.95	42.56	55.43
	3rd Trimester	6	45.50	4.03	1.64	0.17	41.26	49.73

Relation of low-density lipoprotein cholesterol (HDL-C) with preeclampsia, GDM, and preterm

Mean serum LDL-cholesterol level in normal patients was 137.80 mg/dl with an SD of 13.67 mg/dl; in preeclamptic patients, the mean was 137.80 mg/dl with an SD of 11.5 mg/dl. Compared to the normal value of 130 mg/dl [7], the triglyceride level was raised in normal pregnancy. In preeclamptic women, the LDL-C level was the same as in normal pregnancy (Table 4).

**Table 4:** Association of LDL Cholesterol with Preeclampsia, GDM, and Preterm CI: confidence interval; SD: standard deviation, SEM: standard error of the mean; N: number

Outcome	Trimester	N	Mean	SD	SEM	P Value	95% CI	
							Lower Bound	Upper Bound
Preeclampsia	2nd Trimester	20	92.70	18.22	4.07	0.94	84.17	101.23
	3rd Trimester	20	137.80	11.59	2.59	0.99	132.37	143.22
Gestational Diabetes Mellitus	2nd Trimester	6	96.83	31.39	12.81	0.58	63.89	129.77
	3rd Trimester	6	150.16	9.88	4.03	0.02	139.79	160.54
Preterm	2nd Trimester	6	84.50	6.12	2.50	0.30	78.07	90.92
	3rd Trimester	6	127.83	10.64	4.34	0.07	116.66	139.00

There was no significant statistical significance observed between serum LDL-C levels and preeclampsia and preterm in both second and third trimesters. However, a statistical significance was seen between GDM and LDL-C levels in the third trimester but not in the second trimester.

Relation of very low-density lipoprotein cholesterol (VLDL-C) with eclampsia, GDM, and preterm

In the third trimester, the mean serum VLDL-C level in normal patients was 35.88 mg/dl with an SD of 6.5 mg/dl; in preeclamptic patients, the mean was 39.7 mg/dl with an SD of 7.1 mg/dl. Compared to the normal value of 35 mg/dl reported in our previous paper [7], the VLDL-C level was raised in normal pregnancy. In preeclamptic women, the VLDL-C level was increased more than that in normal pregnancy.

In preeclamptic patients, the mean serum VLDL-C levels in the second trimester were 30.9 mg/dl with an SD 7.9 mg/dl (p = 0.93); in the third trimester, the mean was 39.7 mg/dl with an SD of 7.1 mg/dl (p = 0.016). There was no significant statistical significance observed between the serum VLDL-C levels and preeclampsia in the second trimester but significance was found in the third trimesters. There was no statistical significance seen in GDM and preterm with VLDL-C levels in both second and third trimesters.

**Table 5:** Association of VLDL Cholesterol with Eclampsia, GDM, and Preterm CI: confidence interval; SD: standard deviation, SEM: standard error of the mean; N: number

Outcome	Trimester	N	Mean	SD	SEM	P Value	95% CI	
							Lower Bound	Upper Bound
Eclampsia	2nd Trimester	20	30.95	7.93	1.77	0.93	27.23	34.66
	3rd Trimester	20	39.70	7.11	1.59	0.01	36.36	43.03
Gestational Diabetes Mellitus	2nd Trimester	6	27.16	6.01	2.45	0.73	20.85	33.47
	3rd Trimester	6	34.00	5.65	2.30	0.41	28.06	39.93
Preterm	2nd Trimester	6	25.66	3.98	1.62	0.41	21.48	29.84
	3rd Trimester	6	36.83	6.96	2.84	0.84	29.52	44.14

Lipid parameters with outcome in 2nd and 3rd trimesters

**Figure 1:**
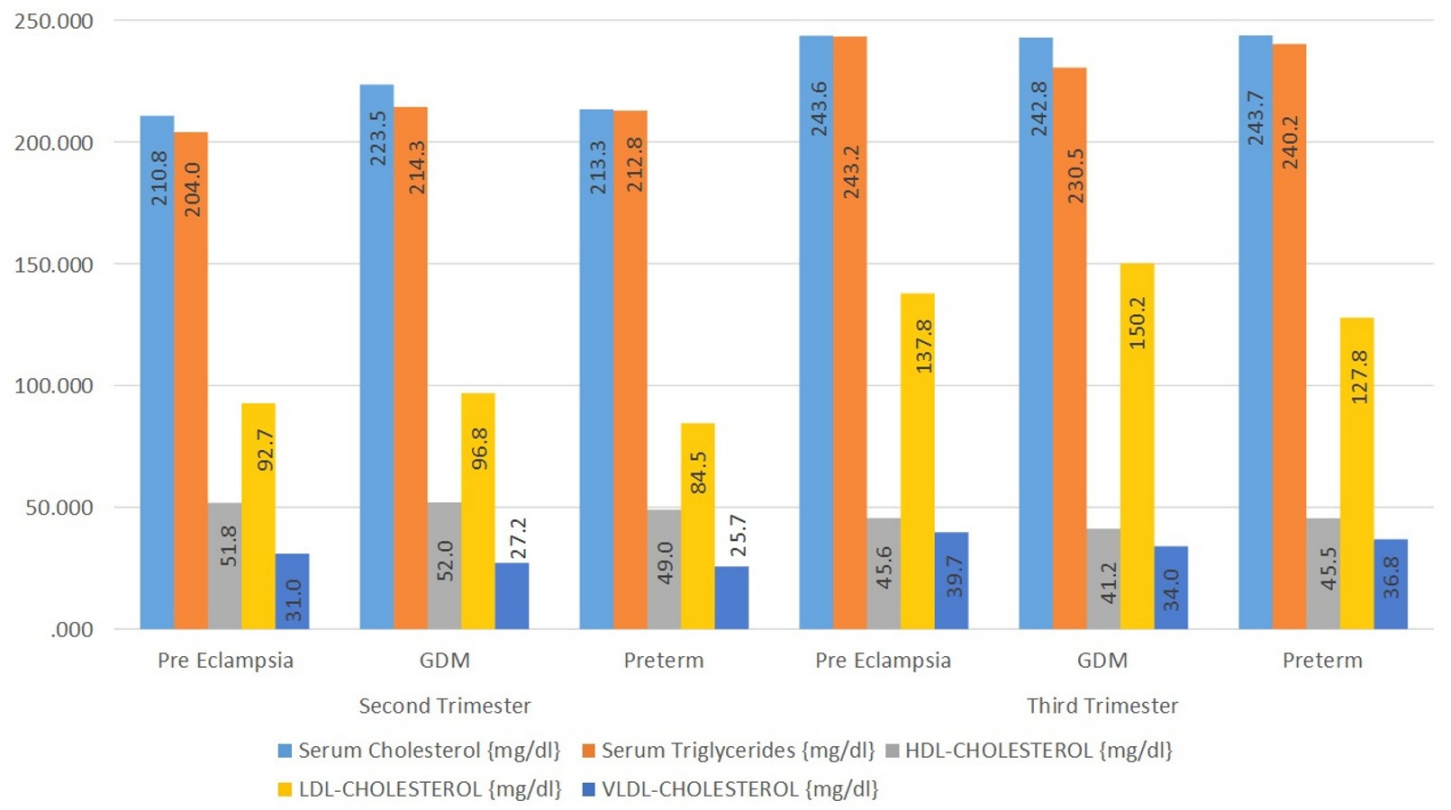
Comparison of lipid parameters between second and third trimesters HDL: high-density lipoprotein; LDL: low-density lipoprotein; VLDL: very-low-density lipoprotein; GDM: gestational diabetes mellitus

The mean values of serum cholesterol, serum TG, HDL-C, LDL-C, and VLDL-C are given in Table 6 and Figure 1.

**Table 6:** Mean Values of Lipid Parameters with Outcome in Second and Third Trimesters HDL: high-density lipoprotein; LDL: low-density lipoprotein; VLDL: very-low-density lipoprotein; GDM: gestational diabetes mellitus

Trimester	Outcome	Serum Cholesterol {mg/dl}	Serum Triglycerides {mg/dl}	HDL-CHOLESTEROL {mg/dl}	LDL-CHOLESTEROL {mg/dl}	VLDL-CHOLESTEROL {mg/dl}
Second Trimester	Preeclampsia	210.75	204.00	51.80	92.70	30.95
	GDM	223.50	214.33	52.00	96.83	27.16
	Preterm	213.33	212.83	49.00	84.50	25.66
Third Trimester	Preeclampsia	243.60	243.20	45.60	137.80	39.70
	GDM	242.83	230.50	41.16	150.16	34.00
	Preterm	243.66	240.16	45.50	127.83	36.83

Complication outcomes

Out of the 200 patients, 168 had no complications, while 20 (10%) had preeclampsia, six (3%) had gestational diabetes mellitus, and the other six (3%) had preterm deliveries (Table 7).

**Table 7:** Distribution According to Complications

Complication	No of Cases	Percentage
No Complication	168	84%
Preeclampsia	20	10%
GDM	6	3%
Preterm	6	3%
Total	200	100%

## Discussion

Hypercholesterolemia is known to cause excessive lipid peroxidation and coexistent diminution in antioxidant activity, which may result in an imbalance between peroxidases and antioxidants, leading to oxidative stress. Oxidative stress and an elevated atherogenic index may lead to atherogenicity in preeclampsia [9].

Triglycerides

In a study conducted by Wiznitzer, et al. to prove the association of lipid levels during gestation with preeclampsia and GDM in 9,911 pregnant women, they observed that the composite endpoint (GDM or preeclampsia) occurred in 1,209 women (12.2%). During the index pregnancy, GDM was diagnosed in 638 women (6.4%), while preeclampsia was diagnosed in 625 pregnancies (6.3%) [10].

In a study by Lorentzen, et al., it was observed that the mean triglyceride concentrations of preeclampsia patients were higher than in normal pregnant women at 16 - 18 weeks [11].

Later, a large prospective cohort study conducted in Norway by Clausen, et al. in 2001 also demonstrated that women with triglycerides above 212 mg/dL (2.4 mmol/L) had a five-fold increased risk (95% CI 1.1 - 23.1) of early-onset preeclampsia (onset before 34 weeks) compared with those with triglycerides levels ≤ 133 mg/dL [12].

A study was done by Niromanesh, et al. to compare the outcomes of 45 pregnant women who had high TG levels (> 195 mg/dl) with 135 pregnant women having TG levels < 195 mg/dl. The important outcome was the incidence of GDM, preterm birth, preeclampsia, and uterine artery pulsatility index. Eight women with high TG levels had preeclampsia (17.8% vs. 3.7% in the control group, p < 0.004), while 11 women had preterm birth (< 37 weeks) (24.4% vs. 5.9% in the control group with OR 5.1, 95% CI 1.9 – 13.8, p < 0.0001). The incidence of GDM in the high triglyceride group was significantly greater than that in the control group. They concluded a positive relation between hypertriglyceridaemia and preeclampsia, preterm birth, and gestational diabetes [13].

In a study done by Kandimalla, et al., 156 pregnant women attending antenatal clinic visits were included prior to 20 weeks and were analysed for lipid levels. One hundred and two subjects out of 156 were followed and monitored for preeclampsia until delivery. The mean TG levels were found to be significantly higher in the preeclampsia group in their report. Women with TG levels above 130 mg/dL had an increased risk of developing preeclampsia compared with those with TG levels of 91 mg/dL or less [14].

From the present study, we can conclude that triglyceride levels of more than 195 mg/dl during the second trimester can lead to complications like preeclampsia. Triglycerides greater than 199.42 mg/dl and 203.24 mg/dl can lead to GDM and preterm delivery, respectively. We can also conclude that a triglyceride level of more than 236 mg/dl during the second trimester can lead to complications like preeclampsia. Triglyceride levels of more than 216.88 mg/dl and 233.98 mg/dl lead to GDM and preterm delivery, respectively, as shown in Table 1. Our findings correlate with the findings of the study done by Kandimalla, et al. [14].

Cholesterol

In a case-control study by Gratacos, et al. in 1996, the total cholesterol levels were not significantly elevated in preeclampsia patients, but triglyceride levels were significantly increased from 10 weeks [15]. Our results correlate with the study done by Wiznitzer, et al., where they concluded high triglyceride levels were associated with the development of gestational diabetes [10]. According to the study done by Adiga, et al., it was concluded that hypercholesterolemia leads to atherogenicity in preeclampsia [16].

From the present study, we cannot predict the occurrence of preeclampsia, GDM, and preterm delivery based on cholesterol levels of the second trimester as the lower bound value is within the normal range. However, we can conclude that cholesterol levels of more than 231.5 mg/dl, 214.35 mg/dl, and 215.12 mg/dl during the second trimester can lead to complications like preeclampsia, GDM, and preterm delivery, respectively, as shown in Table 2.

High-density lipoprotein cholesterol (HDL-C)

In their study, Kandimalla, et al., reported that mean HDL-C levels were found to be lower in the preeclampsia group [14]. In a study done by Wakatsuki, et al., the levels of HDL-C did not differ significantly between preeclamptic women and normal pregnant women [17]. There were other studies as well, which showed a decrease in HDL-C levels during pregnancy [16, 18-20].

From the present study, we cannot predict the occurrence of preeclampsia, GDM, and preterm delivery based on HDL cholesterol levels of the second trimester as the lower bound value and mean are within the normal range. We also cannot predict the occurrence of preeclampsia, GDM, and preterm delivery based on HDL-C levels of the third trimester as the lower bound value and mean are within the normal range as shown in Table 3.

Low-density lipoprotein cholesterol (LDL-C)

Kandimalla, et al. reported mean LDL-C levels were found to be significantly higher in the preeclampsia group [14].

From the present study, we cannot predict the occurrence of preeclampsia, GDM, and preterm delivery based on LDL cholesterol levels of the second trimester as the lower bound value and mean are within the normal range. We cannot predict the occurrence of preeclampsia, GDM, and preterm delivery based on LDL-C levels of the third trimester as the lower bound value and mean are within the normal range as shown in Table 4.

Very low-density lipoprotein cholesterol (VLDL-C)

From the present study, we cannot predict the occurrence of preeclampsia, GDM, and preterm delivery based on VLDL-C levels of the second trimester as the lower bound value and mean are within normal range. We cannot predict the occurrence of preeclampsia, GDM, and preterm delivery based on VLDL-C levels of the second trimester as the lower bound value and mean are within the normal range as seen in Table 5.

Our findings correlate with the findings of a study done by Kandimalla, et al. [14]. Furthermore, high fat and sugar levels may lead to HTN as well and subsequently preeclampsia [21-22].

The findings of this study were similar to the results of these researchers despite confounding variables, such as ethnicity. Our data suggest that the alteration of the lipoprotein metabolism, especially increased triglyceride levels, plays a role in the development of preeclampsia. Because of its early presentation, hypertriglyceridemia can be a useful early predictor of preeclampsia risk. This may help in developing effective early preventive or therapeutic measures.

Limitation of the study

The present study uses the same data from our previous study, which creates a possibility of salome splicing error. However, the present study has different aims, results, and conclusions that were not possible to include in our previous study. Hence, it was pre-decided to publish them differently. 

The strengths of the present study are its prospective nature, assessment of lipid levels in early gestation, and application of strict selection criteria. Some of the limitations are single-centre-based study are the small number of preeclampsia patients and the non-availability of reference serum lipid values in the local population. The way forward will, therefore, be to conduct larger prospective multicentre studies. 

## Conclusions

An association between maternal early pregnancy triglyceridaemia and the subsequent risk of preeclampsia, gestational diabetes mellitus, and preterm deliveries was observed. Triglycerides greater than 195 mg/dl in the second trimester and 236 mg/dl in the third trimester predispose pregnant females to develop preeclampsia. Triglycerides greater than 199 mg/dl in the second trimester and 216 mg/dl in the third trimester predispose pregnant females to develop gestational diabetes. Triglycerides greater than 203 mg/dl in the second trimester and greater than 233 mg/dl in the third trimester predispose pregnant females to have preterm deliveries.

The occurrence of preeclampsia, gestational diabetes mellitus, and preterm deliveries cannot be predicted based on the values of serum cholesterol, HDL cholesterol, LDL cholesterol, and VLDL cholesterol. Fasting blood sugar (FBS) levels greater than 101 mg/dl and postprandial blood sugar (PPBS) greater than 143 mg/dl predispose pregnant females to develop gestational diabetes.

Hence, estimation of lipid profiles during pregnancy is strongly recommended as part of the laboratory investigation so as to instill prompt management strategies to prevent the deleterious effects of hyperlipidaemia associated with pregnancy.

## Appendices

BG designed the entire study. RP wrote the results section and calculated the statistics. VM wrote the first draft. All authors have read the final draft and agreed to publish it.
